# Supplementary material for: Molecular layer interneurons in the cerebellum encode for valence in associative learning
Source: Nat Commun. 2020 Aug 31;11:4217. doi: 10.1038/s41467-020-18034-2 (PMC7459332; doi:10.1038/s41467-020-18034-2)
Supplement: Supplementary file 1 — Supplementary Information [file 41467_2020_18034_MOESM1_ESM.pdf]

Supplementary Information

Molecular layer interneurons in the cerebellum encode for valence in associative learning

Ming Ma et al.

Supplementary Note 1. The dimensionality of the MLI odorant responses is low. The plots in Fig. 1e and Supplementary Fig. 4 show that odorant responses are alike between ROIs in the FOV. This raised the question whether the dimensionality of the MLI responses was small. In order to provide an estimate of the number of independent components comprising the ΔF/F responsiveness of the ensemble we calculated a quantitative measure of dimensionality[1] (see Methods). Supplementary Fig. 6 shows the dimensionality for four sessions with a total number of ROIs ranging from 103 to 136. The dimensionality ranged from 2 to 6 and did not differ significantly between time periods or between naïve and proficient (GLM $p>0.05$, 24 observations, 18 d.f., n=4 sessions, 4 mice, GLM F-statistic=2.42, $p>0.05$). This shows that Ca^{2+} ensemble activity is highly redundant for MLIs.

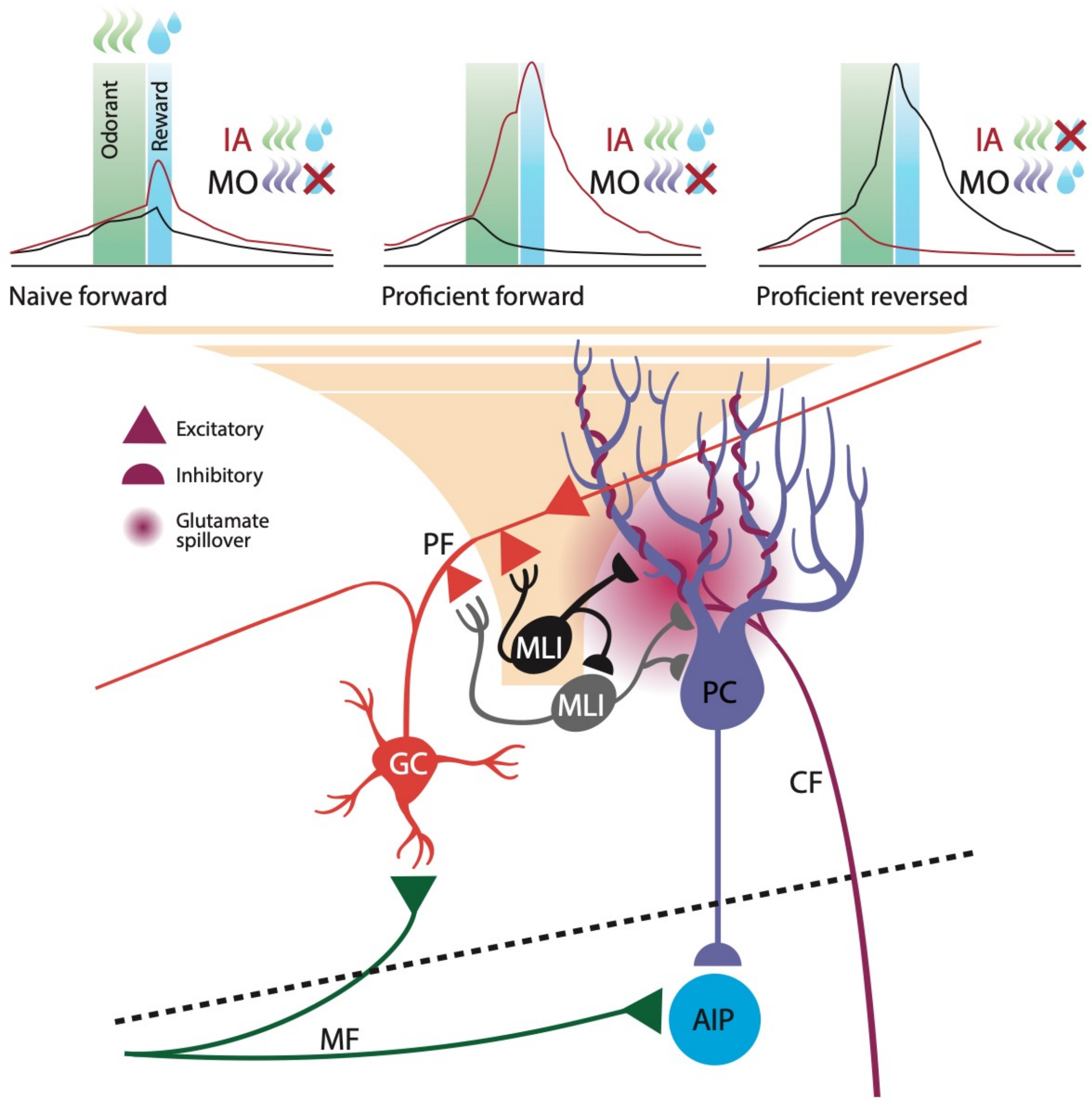

Supplementary Figure 1. Diagram of the cerebellar circuit and summary of the findings. This figure shows the circuit for the cerebellum. Climbing fibers (CFs) innervate the dendrites of Purkinje cells (PCs). Sensorimotor input is conveyed by mossy fibers (MFs) to granule cells (GCs). The GCs innervate the dendrites of the PCs through parallel fibers (PFs). In this project we study the activity of molecular layer interneurons (MLIs) that receive innervation from GCs and inhibit

PCs through a feedforward circuit. However, these neurons have also been proposed to from a disinhibitory motif where an MLI inhibits a deeper MLI that has a high probability of inhibiting PCs[2]. The traces on top represent the ΔF/F time courses recorded from MLIs in the naïve mouse (left), the proficient mouse (center) and the proficient mouse after reversal of the rewarded odorant (right). We found that the activity of the MLIs reflect the valence of the odorants.

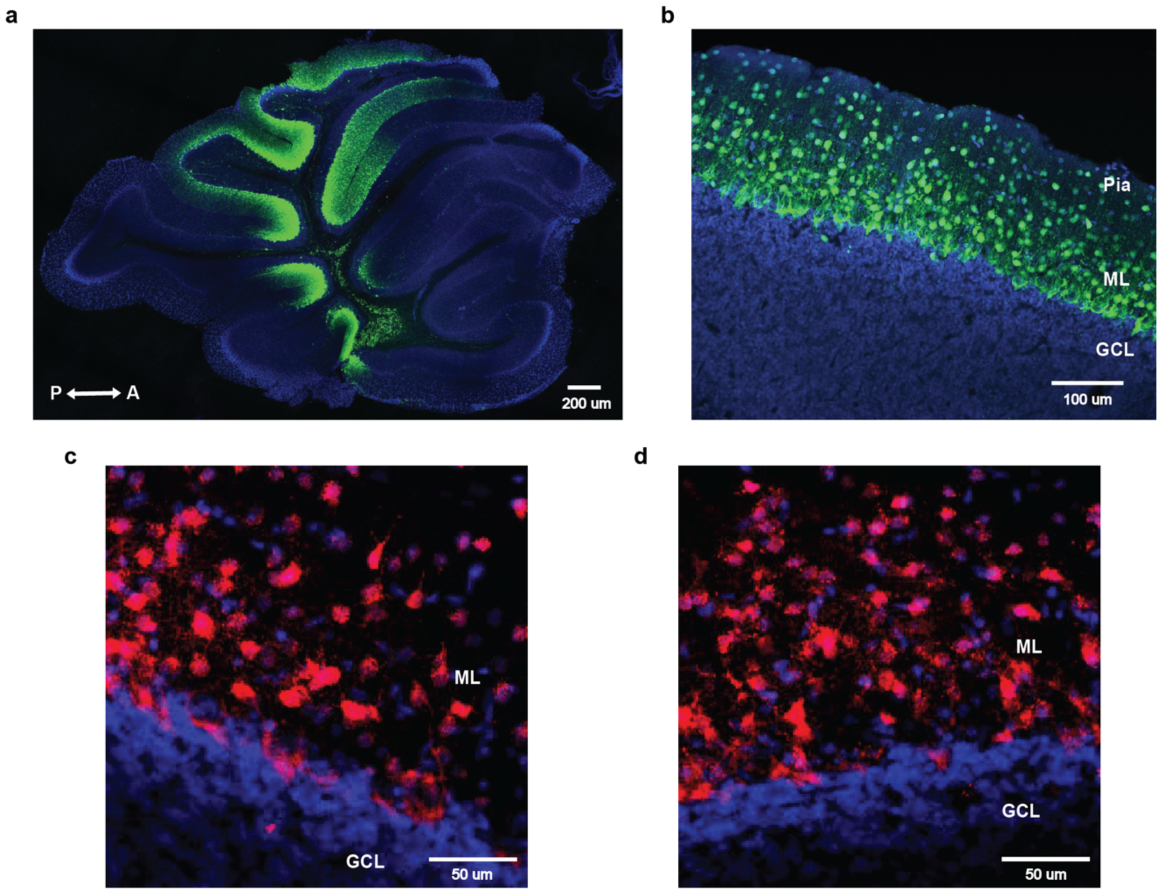

Supplementary Figure 2. Expression of GCaMP6f and mCherry in fixed tissue slices.

a,b. GCaMP6f fluorescence in cerebellar sagittal brain slices from PV-Cre mice infected with AAV1-Syn-Flex-GCaMP6f. GCaMP6f (green) is expressed in the molecular layer. The slices were counterstained with DAPI. This was reproduced in three animals. **c,d.** mCherry fluorescence in cerebellar sagittal brain slices from PV-Cre mice infected with either AAV8-hSyn-DIO-mCherry (**c**) or AAV8-hSyn-DIO-hM4D(Gi)-mCherry (**d**). The slices were counterstained with DAPI. This was reproduced in eight animals.

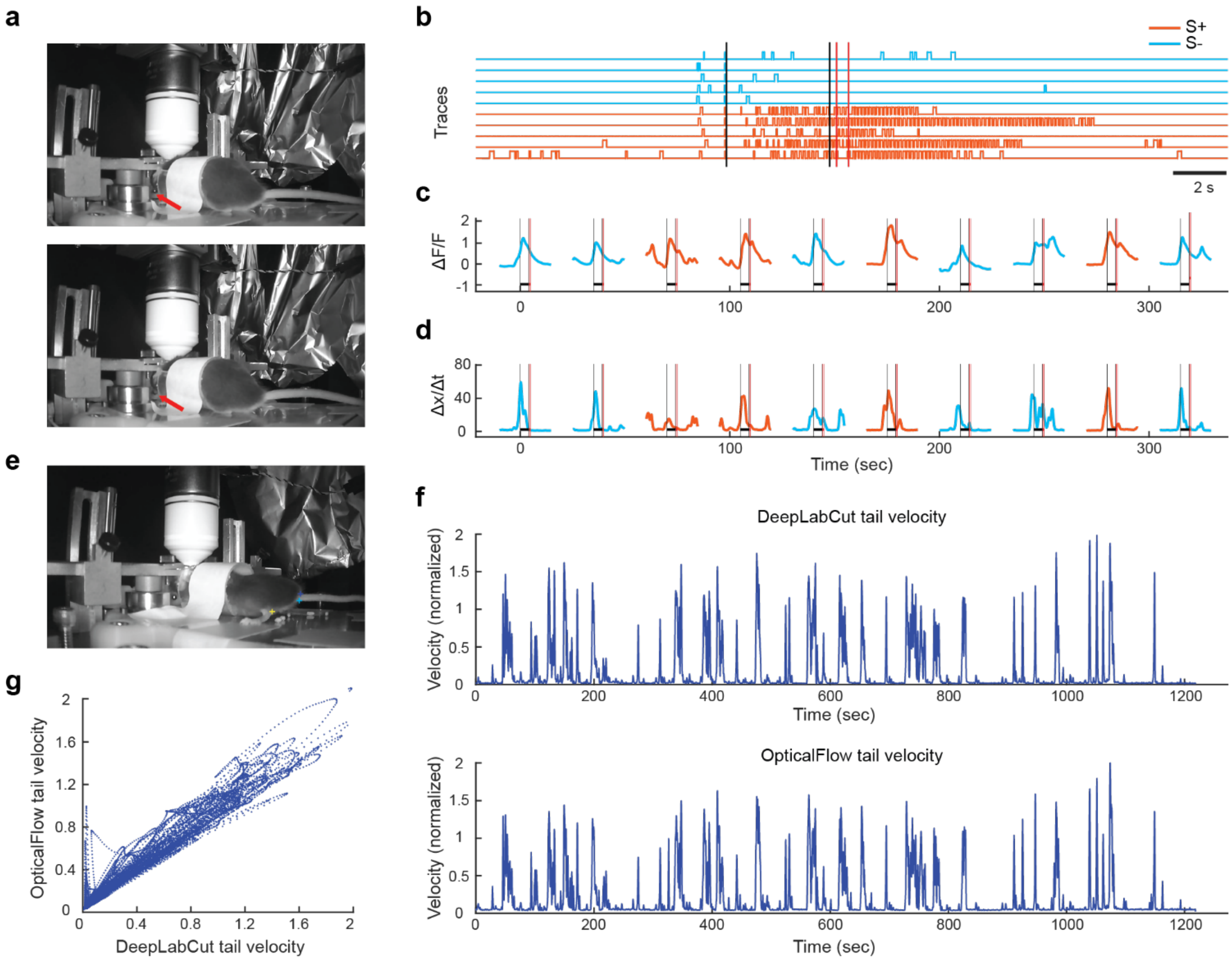

Supplementary Figure 3. The mouse moves the body during the trial. a. Images show the mouse before (upper) and at the start (lower) of the trial. **b.** Lick traces for a subset of trials for a mouse that was proficient (>=80% correct) in the go-no go task. The mouse started the trial by licking on the water spout and the odorant was delivered after a random delay of 1 to 1.5 sec. Orange: S+, light blue: S-. The two vertical lines denote times for odorant on and off (black lines) and reinforcement on and off (red lines). **c.** Examples of ΔF/F Ca^{2+} traces for a subset of trials. **d.** Velocity of mouse movement for these trials measured using optical flow. The vertical black lines are odorant onset and removal and the red lines bound the reinforcement period. **e.** DeepLabCut was used to track the base of the tail. Cross markers show the features tracked. **f.** Comparison of the time course for the velocity of the tail measured with DeepLabCut (upper panel) or optic flow

(lower panel). **g.** Relationship between the velocity of the tail measured with DeepLabCut vs. the velocity of the tail measured with optic flow for all time points in the traces shown in e and f. rho=0.98, $p<0.05$.

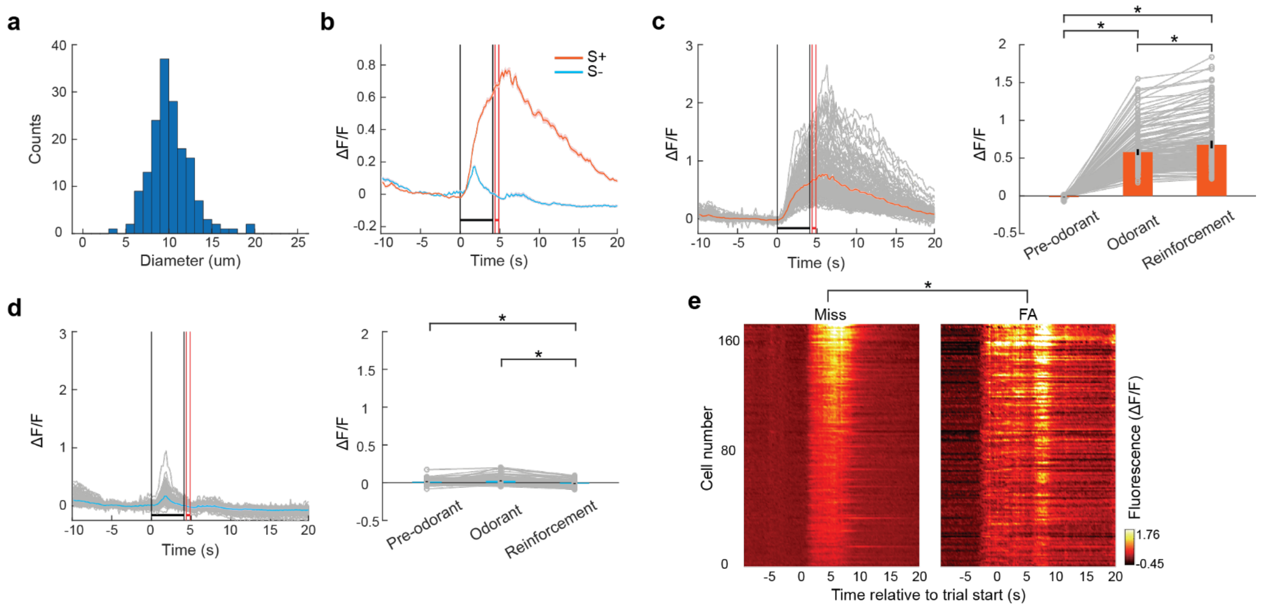

Supplementary Fig. 4. Analysis of changes in ΔF/F for all regions of interest in the time stack for the example shown in Fig. 1. a. Histogram of the average diameter of the ROIs in Fig. 1d. The average diameter was 10.5±4 µm (mean±SD, n=170). **b.** Per trial time course for ΔF/F (mean±95% CI) for the S+ (orange) and S- (light blue) odorants. The vertical black lines are odorant onset and removal and the red lines bound the reinforcement period. **c and d.** Per trial time course for ΔF/F for the S+ (c, left panel) and S- (d, left panel) odorants. The orange and light blue lines are the mean ΔF/F calculated over all ROIs and the grey lines are per ROI time courses. ΔF/F calculated for 1 sec before the odorant (pre-odorant), the last second of odorant application (odorant) and 1.5 seconds after reinforcement (reinforcement) for the S+ (c, right panel) and S- (d, right panel) odorants. GLM analysis involving time periods and different odorants (S+ vs. S-) yielded significant differences for the interactions of reinforcement vs. pre-odorant and odorant vs. pre-odorant with S+ vs. S- ($p<0.001$, 1020 observations, 1014 degrees of freedom, n=170 ROIs, 1 mouse, GLM F-statistic 593, $p<0.001$). *Post-hoc ranksum $p<pFDR=0.043$. **e.** Pseudocolor plots displaying the average per trial ΔF/F time course for FA and Miss for all ROIs in this example.

GLM analysis involving time periods pre-odorant (1 sec before odorant onset), odorant (last second during odorant application) and reinforcement (1.5 seconds after reinforcement) and different events (Hits, Miss, CR and FA) yielded significant differences between reinforcement and pre-odorant ($p<0.001$), between odorant and pre-odorant ($p<0.001$), and between all interactions between these two period pairs and all events ($p<0.01$, 2040 observations, 2028 degrees of freedom, n=170 ROIs, 1 mouse, GLM F-statistic 234, $p<0.001$). *Post-hoc ranksum $p<pFDR=0.048$. Error bars are 95% CIs.

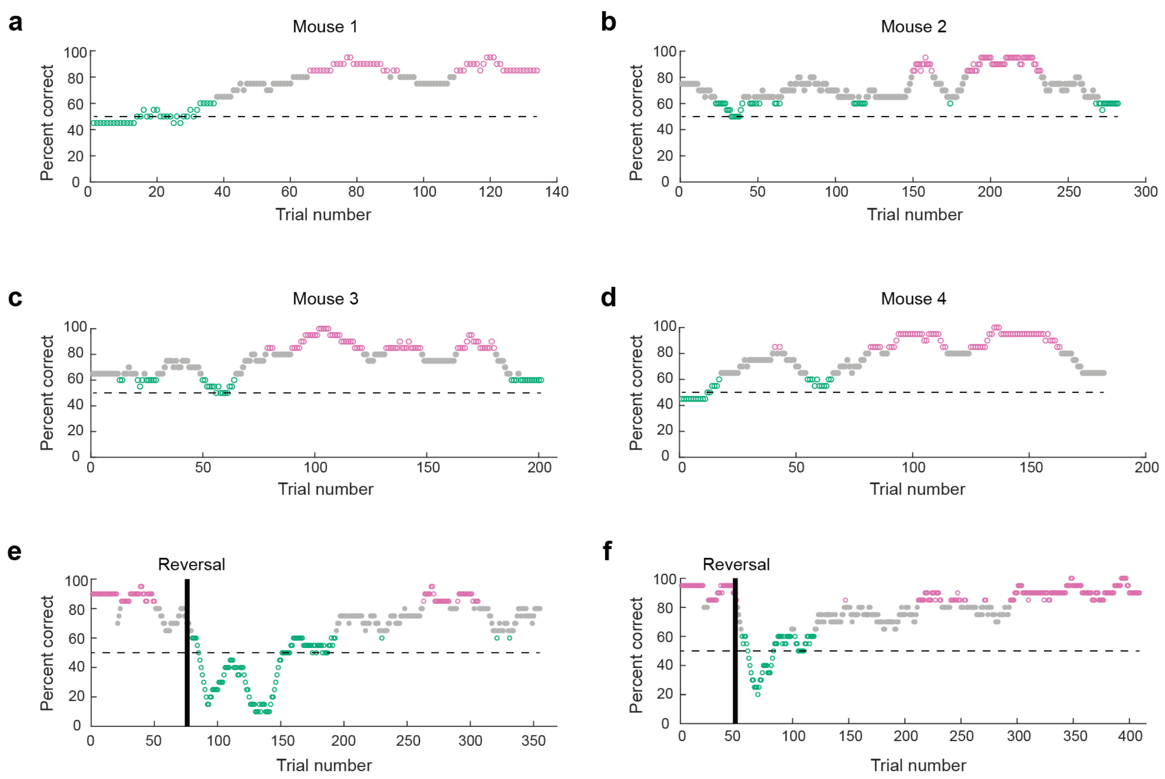

Supplementary Figure 5. Behavioral performance for the mice included in the analysis in Figs. 2i and 4f,h. The plots show percent correct performance for each mouse. Magenta: percent correct >=80%, green percent correct <=65%. **a-d.** Percent correct for mice included in Fig. 2i. **e-f.** Percent correct for two of the three mice included in Figs. 4f and h (the percent correct per trial for the other mouse is shown in Fig. 4a). n=20 trials within a sliding window.

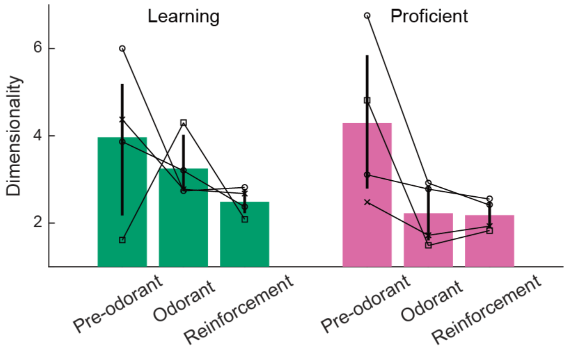

Supplementary Figure 6. Dimensionality for ΔF/F. Dimensionality was calculated in three periods: pre-odorant: 1 sec before odorant onset, odorant: last second of odorant application, reinforcement: 1.5 sec after the onset of water reward. Dimensionality was higher for the pre-odorant period compared to the odorant or reinforcement periods (GLM $p<0.01$, 18 d.f., n=4 sessions, 4 mice). Dimensionality did not differ between naïve and proficient, (GLM $p>0.05$, 24 observations, 18 d.f., n=4 sessions, 4 mice, GLM F-statistic=2.42, $p>0.05$). Error bars are 95% CIs.

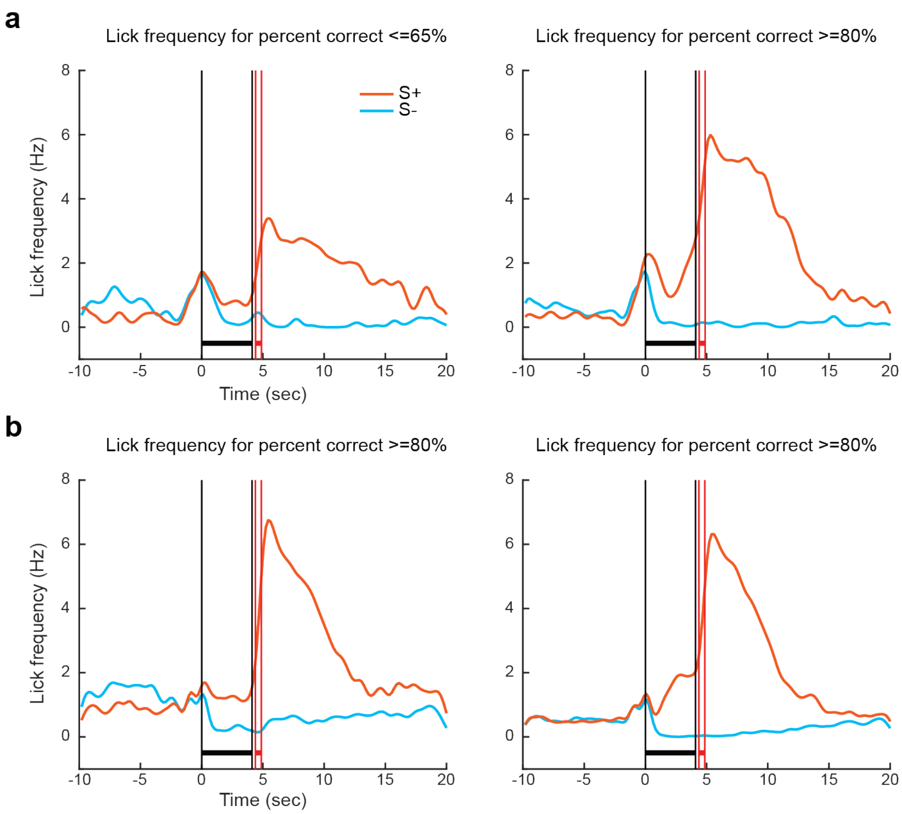

Supplementary Figure 7. Mean lick frequency time courses shown for different experiments. **a.** Lick frequency time courses measured for the experiments whose decoding accuracy time courses are shown in Fig. 3d. **b.** Lick frequency time courses measured for the experiments whose decoding accuracy time courses are shown in Fig. 4g. The vertical black lines are odorant onset and removal and the red lines bound the reinforcement period.

Orange: S+, light blue: S-.

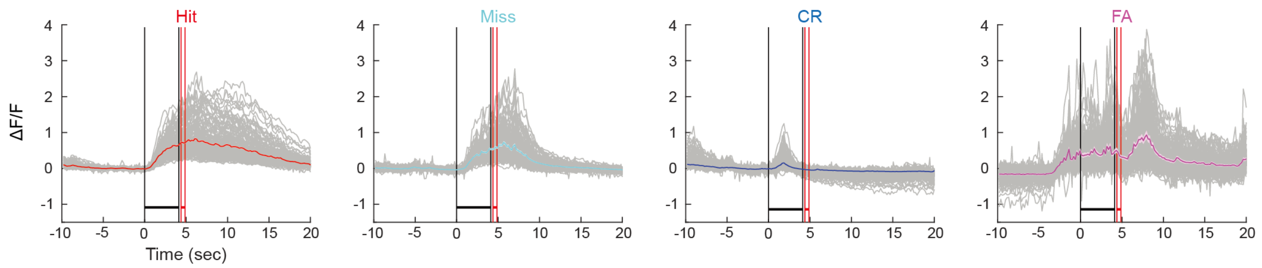

Supplementary Figure 8. Examples of time courses of ΔF/F for different behavioral outcomes in proficient mice. Examples of ΔF/F time courses for 155 ROIs in the FOV shown. The ΔF/F time courses are shown as an average for all trials belonging to specific behavioral outcomes (Hit, Miss, CR or FA) for a single time series.

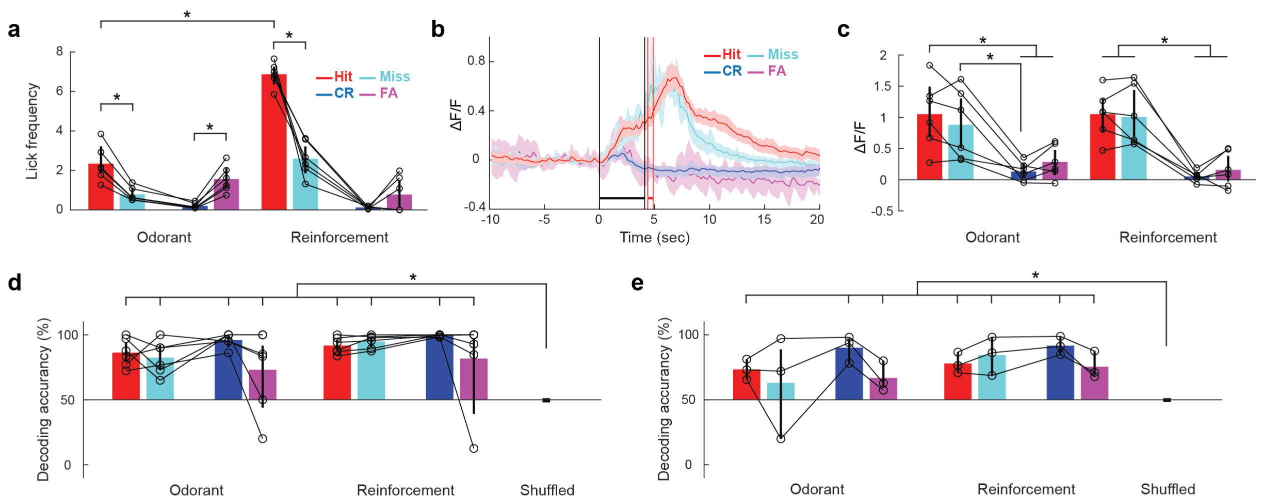

Supplementary Figure 9. Linear discriminant analysis of stimulus identity for error trials for proficient mice. a. Mean lick frequency during the last second of odorant application and the first 1.5 seconds after reward delivery shown for the four trial outcomes. GLM analysis yields a statistically significant difference for odorant vs. reinforcement ($p<0.01$) and for the interaction between odorant vs. reinforcement and CR vs Hit ($p<0.01$) as well as for the interaction between odorant vs. reinforcement and FA vs Hit ($p<0.01$) (48 observations, 40 d.f., n=6 sessions, 5 mice, GLM F-statistic= 68, $p<0.001$). The color denotes the outcome of the trial (Red: Hit, Cyan: Miss, Blue: CR and Magenta: FA). *Post-hoc two sided t test p<pFDR=0.036. **b**. Example of the time course for average ΔF/F (±95% CI, shade) for the four trial outcomes for one session for a mouse performing at percent correct >=80%. The vertical black lines are odorant onset and removal and the red vertical lines bound the reinforcement period. The corresponding lick rate time course is shown in Fig. 5b. **c.** Mean ΔF/F during the last second of odorant application and the first 1.5 seconds after reward delivery shown for the four trial outcomes. GLM analysis yields a statistically significant difference for CR vs Hit ($p<0.001$) as well as for FA vs Hit ($p<0.01$) (48 observations, 40 d.f., n=6 sessions, 5 mice, GLM F-statistic=8.48, $p<0.001$). *Post-hoc two sided t test, p<pFDR=0.027. **d and e.** Decoding accuracy for LDA analysis of stimulus prediction calculated

with $\Delta F/F$ for all ROIs in the FOV classified by trial outcome. **d.** Forward sessions where S+ was Iso and S- was MO. Decoding accuracy differed from shuffled for all outcomes and time periods (two sided t test, $p<pFDR=0.011$, n=6 sessions, 5 mice). GLM did not yield a significant difference for score categories or time period (odorant vs. reinforcement) (48 observations, 40 d.f. $p>0.05$, n=6 sessions, 5 mice, GLM F-statistic=1.49, $p>0.05$). A two-sample *F*-test for equal variances yields a statistically significant difference for the variance between FA and CR ($p<pFDR=0.025$, n=6 sessions, 5 mice). *Post-hoc two sided t test, $p<pFDR=0.01$. **e.** Reverse sessions where S+ was MO and S- was Iso. Decoding accuracy differed from shuffled for all outcomes and time periods (two sided t test, $p<pFDR=0.012$, n=3 sessions, 3 mice). GLM did not yield a significant difference for score categories or time period (odorant vs. reinforcement) (24 observations, 16 d.f. $p>0.05$, n=3 sessions, 3 mice, GLM F-statistic=1.12, $p>0.05$). *Post-hoc two sided t test, $p<pFDR=0.013$. Error bars are 95% CIs.

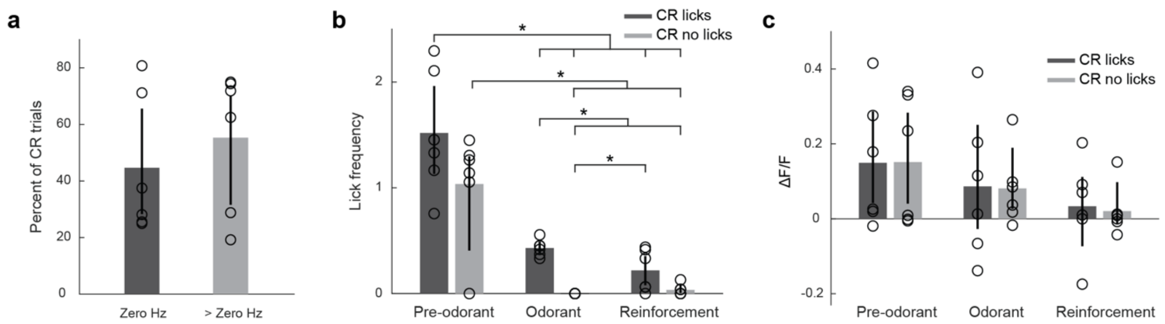

Supplementary Figure 10. Lick frequency and ΔF/F for CR trials with zero licks for proficient mice. The CR trials were classified between trials with no licks in the two 2 second segments for evaluation of lick responses during odorant application (CR-no licks), and other CR trials (CR-licks). **a.** Percent of CR-no licks trials vs. CR-licks trials. A two sided t test does not find a significant difference ($p>0.05$, n=6 sessions, 5 mice). **b.** Lick frequency for CR-no licks and CR-licks for three different periods: one second before odorant application (Pre-odorant), during the two 2 second segments (Odorant) and for 1.5 seconds following reinforcement (Reinforcement). A GLM analysis found a statistically significant difference between CR-licks and CR-no licks ($p<0.05$), and odorant vs. pre-odorant and reinforcement vs. pre-odorant ($p<0.001$, 36 observations, 30 d.f., n=6 sessions, 5 mice, GLM F-statistic=20.3, $p<0.001$). **c.** ΔF/F for the same time periods. A GLM found no differences between the two CR trials and between time periods ($p>0.05$, 36 observations, 30 d.f., n=6 sessions, 5 mice, GLM F-statistic 0.88, $p>0.05$). Error bars are 95% CIs.

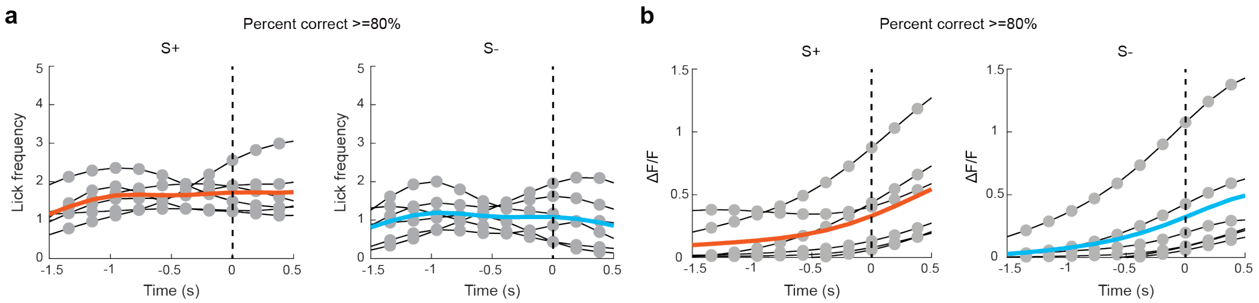

Supplementary Figure 11. Mean ΔF/F and lick frequency time courses for the time period shortly after odorant application when ΔF/F starts increasing for both the S+ and S- odorants. The time courses were aligned to the time after odorant addition when the derivative of ΔF/F increased above 0.03. The data are for six proficient mice. Mean values are shown as a thick orange or light blue line. **a.** Lick frequency calculated as the mean over all trials when the mouse was proficient. In contrast, in this time period there was no increase in lick frequency. GLM analysis yields no significant difference as a function of time, $p>0.05$ and a significant change between S+ and S-, $p<0.001$, 132 observations, 128 d.f., n=6 sessions, 5 mice, GLM F-statistic=21, $p<0.001$. Left panel, S+ odorant. Right panel, S- odorant. **b.** ΔF/F calculated as the mean over all trials when the mouse was proficient. GLM analysis yields a significant change as a function of time, $p<0.05$, and no significant difference between S+ and S-, $p>0.05$, 132 observations, 128 d.f., n=6 sessions, 5 mice, GLM F-statistic = 3.1, $p<0.05$.

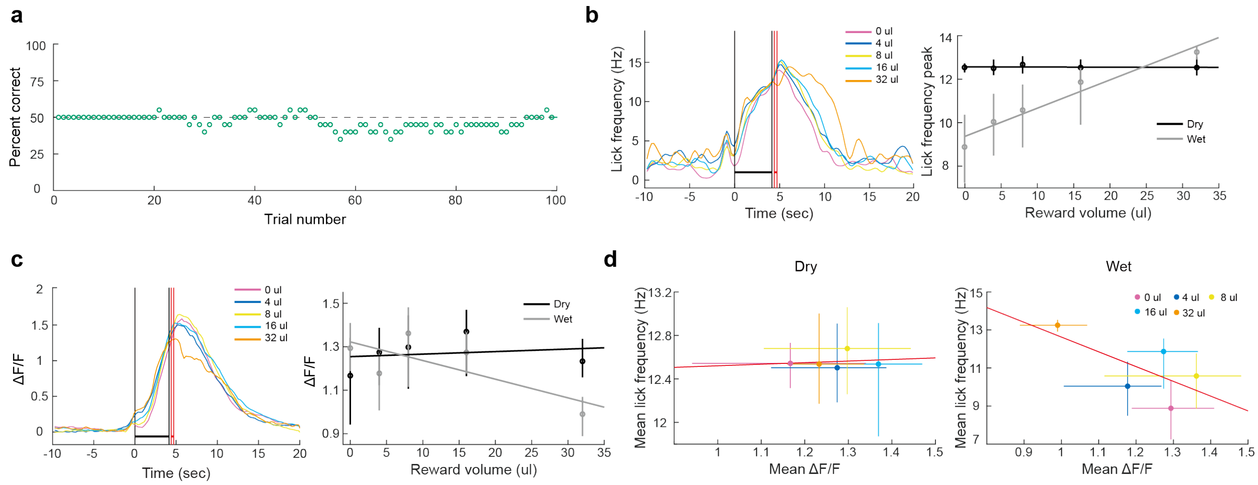

Supplementary Fig. 12. Evaluation of changes in ΔF/F responses for MLIs when the volume of sugar water reward is varied. Results are shown for ΔF/F responses of MLIs and lick frequency for one animal engaged in a go-go task where both odorants were rewarded equally. The volume of sugar water delivered for successful trials was varied from 0 to 32 μl. **a.** Percent correct behavior scored as if this were a go-no go task shows that the mouse responded to both odorants in the go-go task (~50% correct response). n=20 trials within a sliding window. **b**. Left panel: Time course for lick frequency in this go-go experiment. Right panel: Mean per trial lick frequency (±95% CI) calculated at two time points: 4 sec (when the animal is doing dry licks, dry time point) and at 7.5 sec (when the animal is licking to receive the reward, wet time point). GLM analysis yielded a statistically significant difference for lick frequency for dry vs. wet licking and for the interaction between the volume of sugar water delivered and dry vs. wet ($p<0.001$, 198 observations, 194 d.f., 1 session, 1 mouse, GLM F-statistic=21.8, $p<0.001$). **c**. Left panel: Time course for the mean ΔF/F for all trials (averaged for all ROIs per trial in the FOV). Right panel: Mean ΔF/F (±95% CI) calculated at the dry and wet time points. GLM analysis did not yield statistically significant differences ($p>0.05$, 198 observations, 194 d.f., 1 session, 1 mouse, GLM F-statistic=1.7, $p>0.05$). **d.** Relationship between the mean lick frequency and mean ΔF/F for dry

(left) and wet (right) time points. The correlation coefficients were not significant for dry ($\rho=0.16$, $p>0.05$) or wet licking ($\rho=-0.66$, $p>0.05$). Error bars are 95% CIs.

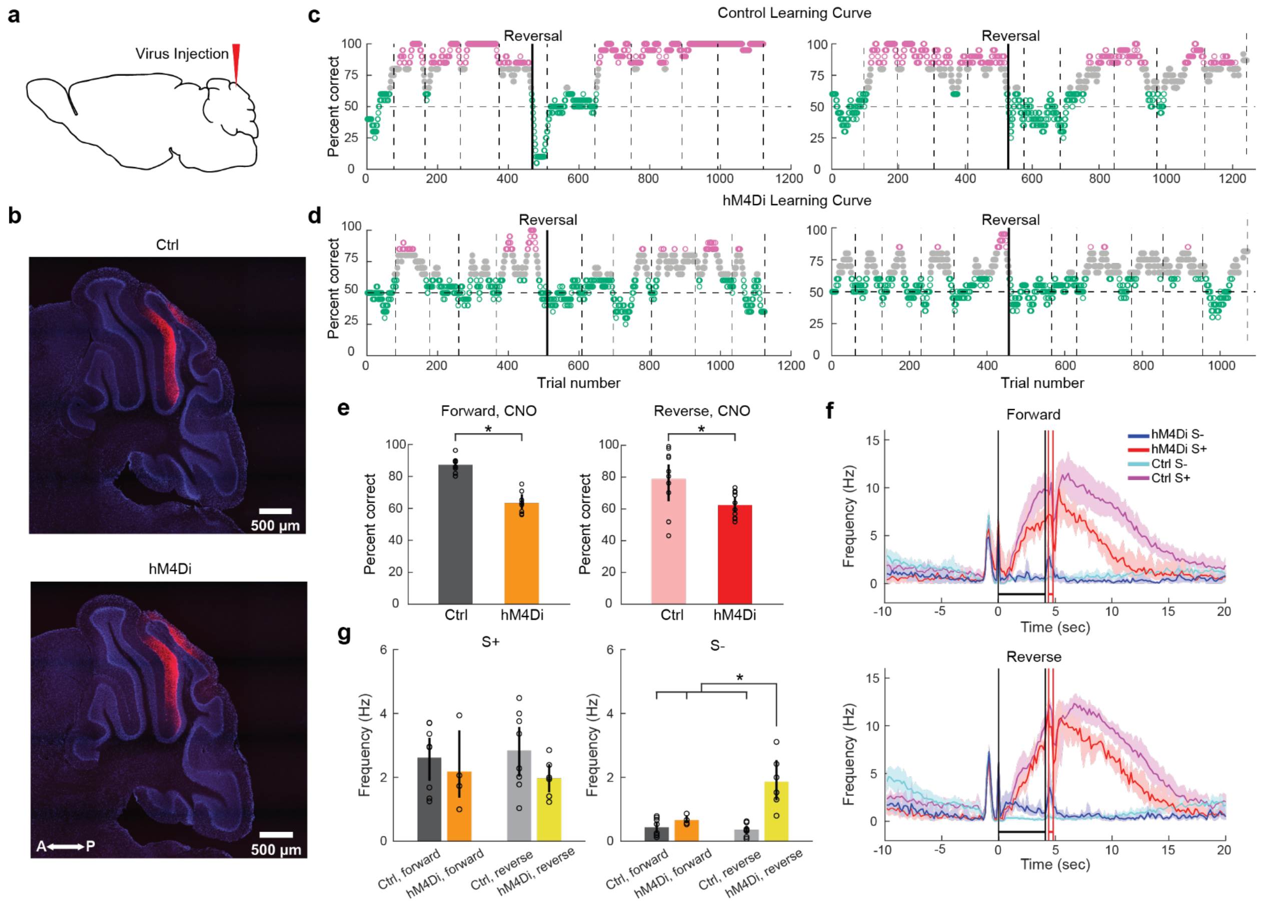

Supplementary Fig. 13. Chemogenetic inhibition of MLI activity impairs associative learning. a. Location of virus injection shown in a sagittal diagram of the brain. **b.** Expression of mCherry and DAPI in the cerebellum 2 months after injection of either AAV8-hSyn-DIO-hM4D(Gi)-mCherry (bottom) or AAV8-hSyn-DIO-mCherry (top) in PV-Cre mice. This was reproduced with two animals. **c. and d.** Examples of behavioral performance in a go-no go task for mice that learned to differentiate between Iso and MO. Green: <=65% correct, magenta: >=80% correct. For these experiments CNO was injected before the start of the session. At the beginning of the experiment (forward session) the rewarded odorant (S+) was 1% iso-amyl acetate and the unrewarded odorant was S-: 1% mineral oil. The reward was reversed at the trial denoted by the vertical line (reversed session). n=20 trials within a sliding window. **c.** Control mice expressing mCherry in MLIs. **d.** Mice expressing hM4Di in MLIs. Left and right panels show the

results of experiments with two separate mice. **e.** Mean percent correct for behavioral performance (mean± 95% CIs, n=4 sessions forward, 5 sessions reversed, 2 mice) for forward (left) and reversed (right) sessions. Note: the first session was excluded because that is usually a low percent session when the animal is learning. A GLM analysis finds a difference between genotypes ($p<0.001$, 36 observations, 32 d.f., 4 sessions for hM4Di and 5 sessions for control, 2 mice, GLM F-statistic=9.3, $p<0.001$). **f**. Average lick frequency time course for forward (top) and reversed (bottom) sessions for mice performing >75% correct (mean± 95% CI, shade, n=4 sessions forward, 5 sessions reversed, 2 mice). The vertical black lines are odorant onset and removal and the red lines bound the reinforcement period. **g**. Mean lick frequency (±CI) during the initial portion of the odorant application period (0.8 to 1.8 sec) for mice performing >75% correct (left: S+, right: S-). GLM analysis did not find a statistically significant difference for treatment or hM4Di expression (or interactions) for S+ ($p>0.05$, 26 observations, 22 d.f., two hM4Di mice and two control mice, 8 sessions, GLM F-statistic=0.92, $p>0.05$), and found a difference for genotype x forward vs. reversed for S- ($p<0.001$, 26 observations, 22 d.f, two hM4Di mice and two control mice, 8 sessions, GLM F-statistic=16.2, $p<0.001$). *$p<pFDR=0.03$ for post-hoc two sided t test. Error bars are 95% CIs.

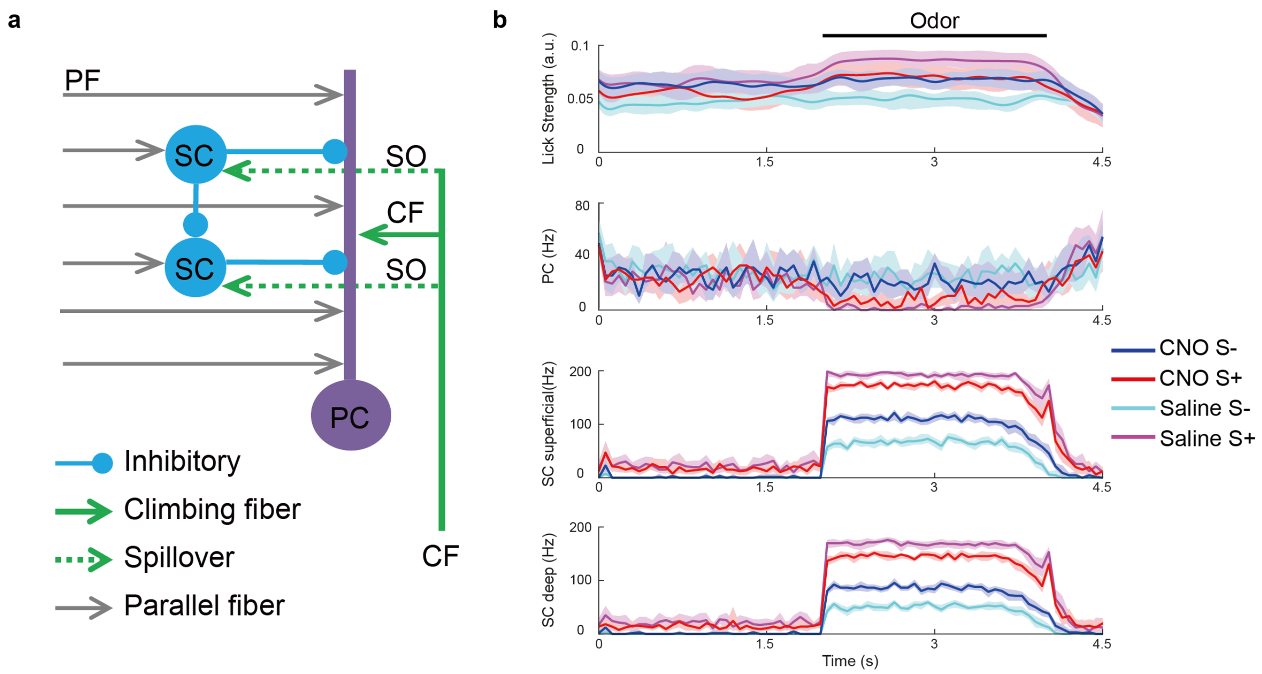

Supplementary Figure 14. Computational simulation of the modulation of PC activity by MLIs. **a.** Schematic representation of the model of the SC/PC circuit. Both SCs and the PC receive excitatory PF inputs. The SCs send inhibitory inputs to the PC. The superficial SC also sends inhibitory inputs to the deep SC. The PC receives strong excitatory inputs from CFs. The SCs also receive excitatory inputs through glutamate spillover from CFs. **b.** Results of odorant stimulation of the neural circuit. The odorant inputs are represented as a sustained increase in the PF firing rate from 2 to 4 seconds. From the top to the bottom, the panels represent lick strength, PC firing rate, and superficial and deep SC firing rates. The model's response for each experimental condition is represented by a specific color. A GLM analysis found that there were significant differences for lick strength for S+ vs S- ($p<0.01$) and CNO ($p<0.05$) and for the interactions between S+ vs. S- and CNO ($p<0.01$, 88 observations, 80 d.f., GLM F-statistic 7.9, $p<0.001$).

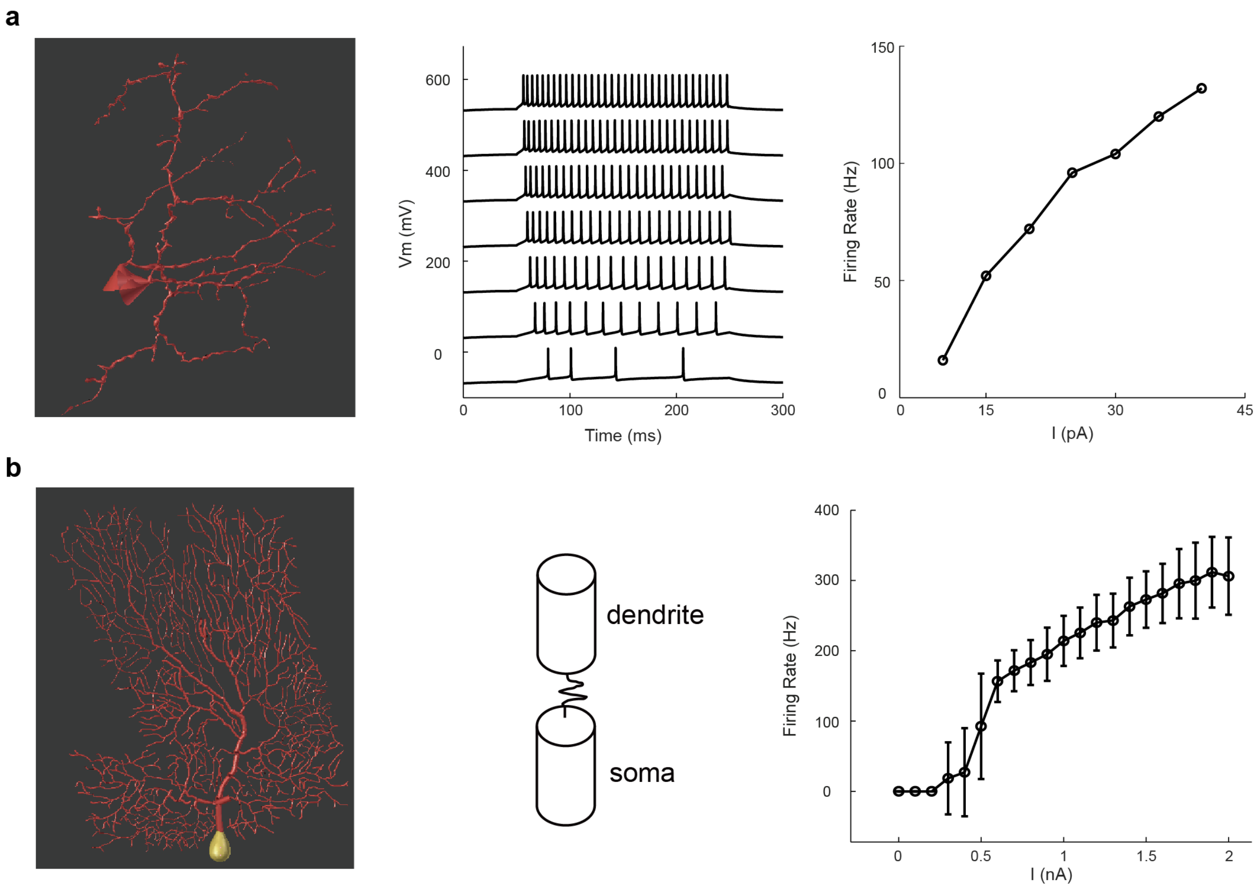

Supplementary Figure 15. Compartmental models of SCs and PC. a. Compartmental model of SC. The model presents the typical firing response of SCs to increasing intensities of a step current of 200 ms. One simulation. **b.** The reduced two compartmental model of PC[3] in the presence of background inhibitory inputs reproduces the typical curve of firing frequency versus input current[4]. The representative morphology of the PC was generated according to Martone and colleagues[5]. Error bars are standard deviation. n=51 simulations.

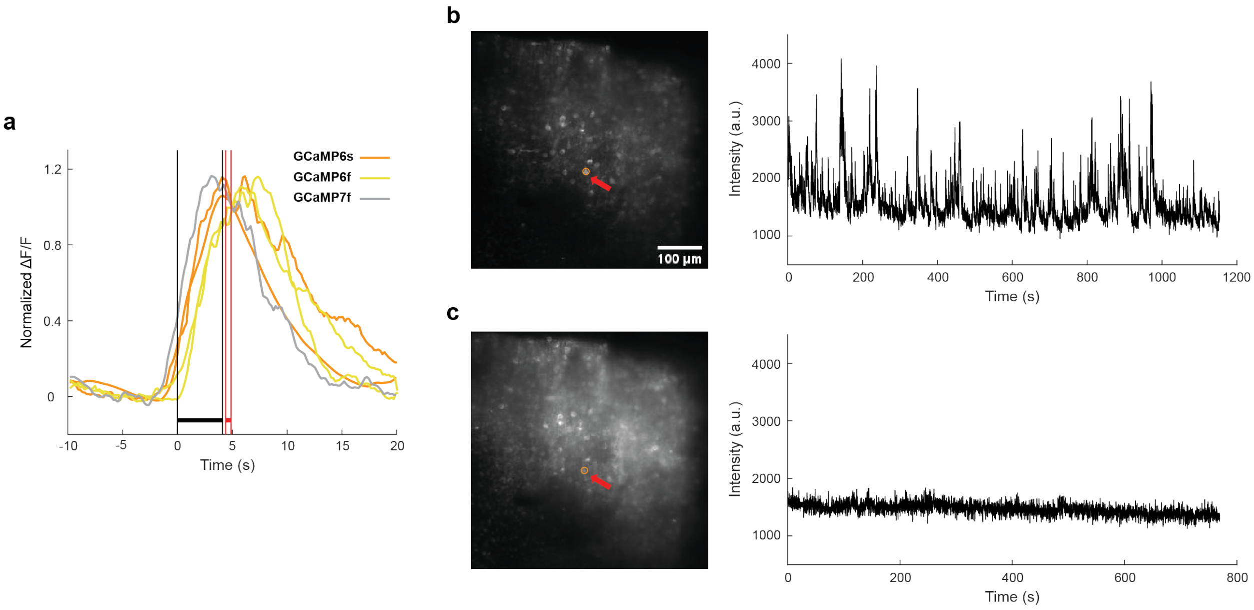

Supplementary Figure 16. Controls for ΔF/F measurements of MLI cytoplasmic Ca^{2+} with GCaMP in the animal engaged in the go-no go task. a. Time courses for average ΔF/F measured for rewarded odorant trials in MLIs of five proficient mice expressing different GCaMP proteins. **b and c.** Control showing that the changes in ΔF/F are not due to axial movements of the ROIs. Two photon imaging was performed in the molecular layer of a mouse engaged in the go-no go task with excitation at either 920 nm (**b**, GCaMP6f emission Ca^{2+}-sensitive) or 820 nm (**c**, GCaMP6f emission Ca^{2+}-insensitive). Fluorescence transients were detected at 920 nm excitation, but not at 820 nm excitation indicating that the transients were not due to axial movement.

Supplementary References

1 Litwin-Kumar, A., Harris, K. D., Axel, R., Sompolinsky, H. & Abbott, L. F. Optimal Degrees of Synaptic Connectivity. *Neuron* **93**, 1153-1164 e1157, doi:10.1016/j.neuron.2017.01.030 (2017).

2 Arlt, C. & Hausser, M. Microcircuit Rules Governing Impact of Single Interneurons on Purkinje Cell Output In Vivo. *Cell Rep* **30**, 3020-3035 e3023, doi:10.1016/j.celrep.2020.02.009 (2020).

3 Forrest, M. D. Simulation of alcohol action upon a detailed Purkinje neuron model and a simpler surrogate model that runs >400 times faster. *BMC Neurosci* **16**, 27, doi:10.1186/s12868-015-0162-6 (2015).

4 Llinas, R. & Sugimori, M. Electrophysiological properties of in vitro Purkinje cell somata in mammalian cerebellar slices. *J Physiol* **305**, 171-195, doi:10.1113/jphysiol.1980.sp013357 (1980).

5 Martone, M. E. *et al.* The cell-centered database: a database for multiscale structural and protein localization data from light and electron microscopy. *Neuroinformatics* **1**, 379-395, doi:10.1385/NI:1:4:379 (2003).
