# Supplementary material for: Molecular layer interneurons in the cerebellum encode for valence in associative learning
Source: Nat Commun. 2020 Aug 31;11:4217. doi: 10.1038/s41467-020-18034-2 (PMC7459332; doi:10.1038/s41467-020-18034-2)
Supplement: Supplementary file 3 — Reporting Summary [file 41467_2020_18034_MOESM3_ESM.pdf]

nature research

Corresponding author(s): Diego Restrepo

Last updated by author(s): Jul 10, 2020

Reporting Summary

Nature Research wishes to improve the reproducibility of the work that we publish. This form provides structure for consistency and transparency in reporting. For further information on Nature Research policies, see Authors & Referees and the Editorial Policy Checklist .

Statistics

For all statistical analyses, confirm that the following items are present in the figure legend, table legend, main text, or Methods section.

n/a	Confirmed	
☐	☒	The exact sample size (*n*) for each experimental group/condition, given as a discrete number and unit of measurement
☐	☒	A statement on whether measurements were taken from distinct samples or whether the same sample was measured repeatedly
☐	☒	The statistical test(s) used AND whether they are one- or two-sided<br>*Only common tests should be described solely by name; describe more complex techniques in the Methods section.*
☐	☒	A description of all covariates tested
☐	☒	A description of any assumptions or corrections, such as tests of normality and adjustment for multiple comparisons
☐	☒	A full description of the statistical parameters including central tendency (e.g. means) or other basic estimates (e.g. regression coefficient) AND variation (e.g. standard deviation) or associated estimates of uncertainty (e.g. confidence intervals)
☐	☒	For null hypothesis testing, the test statistic (e.g. F, t, r) with confidence intervals, effect sizes, degrees of freedom and P value noted<br>*Give P values as exact values whenever suitable.*
☒	☐	For Bayesian analysis, information on the choice of priors and Markov chain Monte Carlo settings
☒	☐	For hierarchical and complex designs, identification of the appropriate level for tests and full reporting of outcomes
☒	☐	Estimates of effect sizes (e.g. Cohen's *d*, Pearson's *r*), indicating how they were calculated

Our web collection on statistics for biologists contains articles on many of the points above.

Software and code

Policy information about availability of computer code

Data collection	Intan software RHD2000, Matlab 9.6
Data analysis	Matlab 9.6, ImageJ 1.52, NLMorphologyViewer 0.3.0, NEURON 7.5, Blender 2.78, addon NeuromorphoVis 1.4.0.

For manuscripts utilizing custom algorithms or software that are central to the research but not yet described in published literature, software must be made available to editors/reviewers. We strongly encourage code deposition in a community repository (e.g. GitHub). See the Nature Research guidelines for submitting code & software for further information.

Data

Policy information about availability of data

All manuscripts must include a data availability statement. This statement should provide the following information, where applicable:

- Accession codes, unique identifiers, or web links for publicly available datasets
- A list of figures that have associated raw data
- A description of any restrictions on data availability

The raw and processed data were uploaded to GigaDB and would be available before publication. The codes used for this study could also be found at github: https://github.com/restrepd/CaImAnDR, with full instructions on how the figures were generated.

Field-specific reporting

Please select the one below that is the best fit for your research. If you are not sure, read the appropriate sections before making your selection.

☐ Life sciences ☒ Behavioural & social sciences ☐ Ecological, evolutionary & environmental sciences

Behavioural & social sciences study design

All studies must disclose on these points even when the disclosure is negative.

Study description	Mice were water deprived by restricting daily consumption of water to 1-1.5 ml. Mice were monitored for signs of dehydration or a decrease in body weight below 80% of the initial weight. When the animals were ready, they were trained in a head-fixed olfactory go-no go task with (1% iso-amyl acetate vs mineral oil odorant application). Licks were monitored by an olfactometer that controlled valves to deliver a 1:40 dilution of odorant at a rate of 2 lt/min. The water-deprived mice started the trial by licking on the water port. In S+ trials, the mice needed to lick at least once in two 2 sec time periods to obtain a reward (0.1 g/ml sucrose water). In S- trials, the mice need to refrain licking one of the two 2 sec segments to avoid a longer inter-trial interval (10 sec). We imaged the cerebellum MLIs during the learning process and the animal's behavior performance was evaluated in a sliding window of 20 trials (10 for S+ and S- trials each, presented in random order). The percent correct value represents the percent of trials in which the animal successfully performed appropriate actions(>=80% is considered as proficient). In reverse go-no go training sessions, the rewarded and un-rewarded odorants were switched. In behavior training, the animals were injected with control or chemogenetic hM4Di virus and underwent different treatment and task conditions to compare the behavior performance. Data are quantitative.
Research sample	All animal procedures were performed in accordance with protocols approved by the Institutional Animal Care and Use Committee of the University of Colorado Anschutz Medical Campus. Mice were bred in the animal facility and we used both 2-6 months old male and female adult Parvalbumin-Cre (PV-Cre) mice and wild-type C57BL/6J mice for experiments. The study sample is representative and we used mice because of the significant amount of information available on cerebellar function in this animal model and the availability of gene-targeted mice.
Sampling strategy	Data was collected for all mice and data comparisons were made within for different conditions (e.g. naive mice vs. proficient mice; CNO treated vs non-treated). We did not know the effect size before doing the experiments. The effect size was large enough and did not require additional experiments. We tested a subset of animals (random sampling). The signal to noise ratio of the changes was large justifying the sample numbers used.
Data collection	The animals were first habituated to the setup to minimize stress for the imaging experiments. The imaging sessions started at least 10 minutes after mice had been head-fixed. We searched for active MLIs while imaging zones in the vermis of lobule VI, between the midline and the paravermal vein. The two photon imaging system consisted of a movable objective microscope (MOM, Sutter Inc) paired with a 80 MHz, ~100 femtosecond laser (Mai-Tai DeepSee, Spectra Physics, USA) centered at 920 nm. The MOM was fitted with a single photon epifluorescence eGFP filter path (475 nm excitation/500-550 nm emission) used for initial field targeting followed by switching to the two photon laser scanning path for imaging GCaMP at the depth of the MLIs. The galvometric laser scanning system was driven by SlideBook 6.0 (Intelligent Imaging Innovations, Inc). The two photon time lapses were acquired at 256 x 256 pixels using a 1.0 NA/20x water emersion objective (Zeiss, Germany) at around 5.3 Hz. On the day of initial imaging, a field of view was selected to image a large number of active cerebellar neurons located in the most superficial planes of the molecular layer likely including mostly stellate cells, and several batches of 6000 frames were be collected in each training session. After two photon imaging a second image of the vasculature was captured wide field epifluorescence to reconfirm the field. For behavior experiments, the animals were injected with either control or chemogenetic hM4Di virus. The animals were trained to perform associative tasks with different treatment and task conditions and licks were recorded using Intan software. All image acquisition, behavior and data analysis was performed by a computer and no data were discarded.
Timing	Imaging and behavior experiments started around one month after the AAV virus was injected, and the imaging and behavior period typically last around 1-2 months.
Data exclusions	No data were excluded
Non-participation	No participants were dropped
Randomization	The animals were randomly chosen for experiments

Reporting for specific materials, systems and methods

We require information from authors about some types of materials, experimental systems and methods used in many studies. Here, indicate whether each material, system or method listed is relevant to your study. If you are not sure if a list item applies to your research, read the appropriate section before selecting a response.

Materials & experimental systems

n/a	Involved in the study	
x		Antibodies
x		Eukaryotic cell lines
x		Palaeontology
	x	Animals and other organisms
x		Human research participants
x		Clinical data

Methods

n/a	Involved in the study	
x		ChIP-seq
x		Flow cytometry
x		MRI-based neuroimaging

Animals and other organisms

Policy information about studies involving animals; ARRIVE guidelines recommended for reporting animal research

Laboratory animals	Both male and female mice of Parvalbumin-Cre (PV-Cre, Jackson Labs: 008069) mice and wild-type C57BL/6J mice, aging between 2-6 months were used for experiment purpose.
Wild animals	No wild animals were used.
Field-collected samples	No field collected samples were used in this study.
Ethics oversight	University of Colorado Anschutz Medical Campus Institutional Animal Care and Use Committee

Note that full information on the approval of the study protocol must also be provided in the manuscript.
