# Supplementary material for: Molecular layer interneurons in the cerebellum encode for valence in associative learning
Source: Nat Commun. 2020 Aug 31;11:4217. doi: 10.1038/s41467-020-18034-2 (PMC7459332; doi:10.1038/s41467-020-18034-2)
Supplement: Supplementary file 4 — Description of Additional Supplementary Files [file 41467_2020_18034_MOESM4_ESM.pdf]

Description of Additional Supplementary Files

File Name: Supplementary Movie 1
Description: The movie shows mouse movement for 29 seconds starting ~10 seconds before trial initiation.

File Name: Supplementary Movie 2
Description: The movie shows changes in GCaMP6f fluorescence for the MLIs in Fig. 1b.
