# Supplementary figures and images for: Molecular layer interneurons in the cerebellum encode for valence in associative learning
Source: Nat Commun. 2020 Aug 31;11:4217. doi: 10.1038/s41467-020-18034-2 (PMC7459332; doi:10.1038/s41467-020-18034-2)

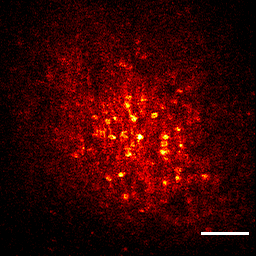

Supplement: Supplementary file 6 — Supplementary Movie 2 [file 41467_2020_18034_MOESM6_ESM.gif]
